# A nanocomposite-based electrochemical sensor for non-enzymatic detection of hydrogen peroxide

**DOI:** 10.18632/oncotarget.14308

**Published:** 2017-01-04

**Authors:** Xin Du, Yuan Chen, Wenhao Dong, Bingkai Han, Min Liu, Qiang Chen, Jun Zhou

**Affiliations:** ^1^ Institute of Biomedical Sciences, College of Life Sciences, Key Laboratory of Animal Resistance Biology of Shandong Province, Key Laboratory of Molecular and Nano Probes of the Ministry of Education, Shandong Normal University, Jinan, Shandong 250014, China; ^2^ State Key Laboratory of Medicinal Chemical Biology, Key Laboratory of Bioactive Materials of the Ministry of Education, College of Life Sciences, Nankai University, Tianjin 300071, China

**Keywords:** hydrogen peroxide, electrochemical sensor, graphene oxide, polyaniline, platinum nanoparticle

## Abstract

Hydrogen peroxide (H_2_O_2_) plays important signaling roles in normal physiology and disease. However, analyzing the actions of H_2_O_2_ is often impeded by the difficulty in detecting this molecule. Herein, we report a novel nanocomposite-based electrochemical sensor for non-enzymatic detection of H_2_O_2_. Graphene oxide (GO) was selected as the dopant for the synthesis of polyaniline (PANI), leading to the successful fabrication of a water-soluble and stable GO-PANI composite. GO-PANI was subsequently subject to cyclic voltammetry to generate reduced GO-PANI (rGO-PANI), enhancing the conductivity of the material. Platinum nanoparticles (PtNPs) were then electrodeposited on the surface of the rGO-PANI-modified glassy carbon electrode (GCE) to form an electrochemical H_2_O_2_ sensor. Compared to previously reported sensors, the rGO-PANI-PtNP/GCE exhibited an expanded linear range, higher sensitivity, and lower detection limit in the quantification of H_2_O_2_. In addition, the sensor displayed outstanding reproducibility and selectivity in real-sample examination. Our study suggests that the rGO-PANI-PtNP/GCE may have broad utility in H_2_O_2_ detection under physiological and pathological conditions.

## INTRODUCTION

Hydrogen peroxide (H_2_O_2_) is a signaling molecule critically involved in various physiological and pathological processes, such as cell migration, cell proliferation, immune response, and circadian rhythm [1, 2]. However, the difficulty of detecting H_2_O_2_ has been an obstacle in investigating its involvement in health and disease. Over the past few decades, a myriad of methods for analytical quantification of H_2_O_2_ have been developed, based on mass spectrometry [3], fluorescence [4], and chemiluminescence [5]. In addition, electrochemical sensors have been developed for the determination of H_2_O_2_. However, most of the electrochemical sensors are based on enzymes and fraught with issues that include low reproducibility and high instability, because enzymes require specific environmental conditions to maintain their activity [6]. Thus, the preparation of non-enzymatic sensors for detecting H_2_O_2_ is believed to have broader applications.

Polyaniline (PANI) is a light-weight, conductive polymer that has been used to modify electrode surfaces due to its excellent electrochemical activity, biocompatibility and low production cost [7–9]. For example, Zhang et al. successfully detected NO^3-^ using a PANI-platinum nanoparticle (PtNP)-coated electrode [10]. Similarly, Naim et al. used thermal annealing to prepare a PANI-Ag-Fe nanocomposite thin film that served as an electrochemical E. coli sensor [11]. PANI-based sensors work well under neutral pH conditions; however, the conductivity and electrochemical activity of PANI is strongly inhibited in solutions with pH values higher than 6 [12]. Moreover, the low cycle time of pure PANI also limits its applications. It has been shown that introducing additional functional groups or dopants can greatly enhance the conductivity and stability of PANI for the purpose of electrode modification [13, 14].

In comparison to other carbon-based materials, the hydrophilic oxygenous group of graphene oxide (GO) results in increased specific surface area and improved water solubility compared to carbon nanotubes. GO could be selected as the dopant for the synthesis of PANI, because it can not only serve as an electrochemical and mechanical supporting material, but can also supply active sites for PANI polymerization. A similar synthetic method was used to prepare supercapacitor electrodes with graphene/polyaniline nanofiber composites [15]. Previous studies have shown that PtNPs can catalyze H_2_O_2_ reduction and decrease the oxidation/reduction overvoltage in H_2_O_2_ detection [16, 17], a critical factor for avoiding interference from other substances present in solution [18, 19]. Thus, integration of PtNPs into reduced GO-PANI (rGO-PANI) composites can improve synthetic methods, while also enhancing the performance of the material in non-enzymatic H_2_O_2_ analysis.

In the present study, we developed a novel nanocomposite-based sensor for non-enzymatic detection of H_2_O_2_, by electrodepositing PtNPs on the surface of the rGO-PANI-coated glassy carbon electrode (GCE). The rGO-PANI-PtNP/GCE had significant advantages over previously reported H_2_O_2_ sensors, both in electrochemical properties and in real-sample detection. To the best of our knowledge, this study is the first to demonstrate the successful production of a functional, non-enzymatic H_2_O_2_ sensor using the rGO-PANI-PtNP nanocomposite.

## RESULTS AND DISCUSSION

### Characterization of the composite materials

**Figure 1:**
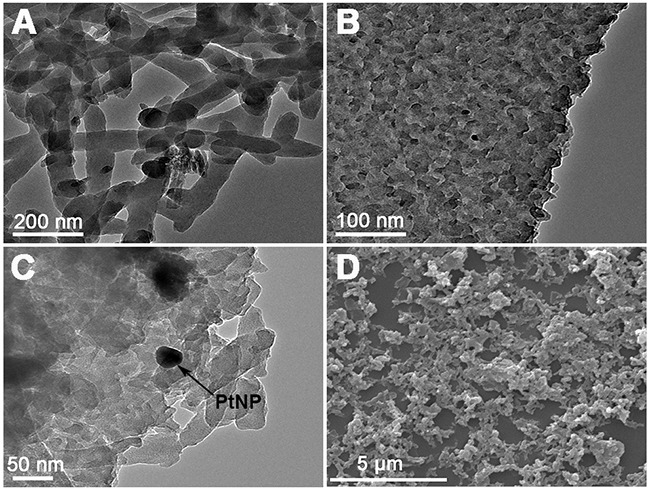
Characterization of the morphology and structure of the composites **(A–C)** TEM and **(D)** SEM images of PANI **(A)**, rGO-PANI **(B and D)** and rGO-PANI-PtNPs **(C)**.

**Scheme 1:**
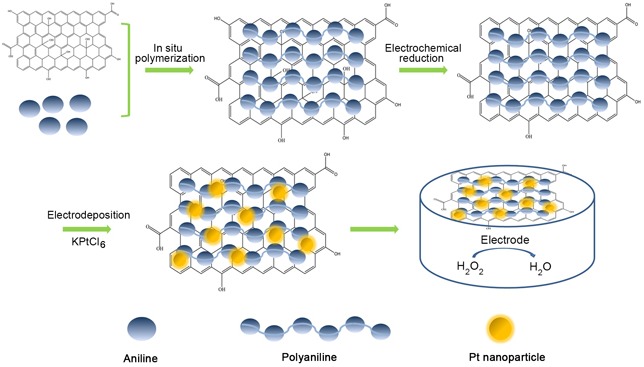
The fabrication process of the rGO-PANI-PtNP/GCE

The rGO-PANI-PtNP/GCE H_2_O_2_ sensor was prepared by electrodepositing PtNPs on the surface of the rGO-PANI-coated GCE (Scheme 1). The morphology and structure of the PANI, rGO-PANI, and rGO-PANI-PtNP composites were characterized with transmission electron microscopy (TEM) and scanning electron microscopy (SEM). TEM revealed that PANI formed uniform fibrous structures of about 50 nm in width and hundreds of nanometers in length (Figure 1A). In comparison to PANI, the structure of rGO-PANI was substantially different (Figure 1B), with the surface of rGO sheets almost completely covered by the fibrous PANI, creating a multilayered structure. PtNPs had a diameter of approximately 30 nm and decorated the surface of rGO-PANI (Figure 1C). We then performed SEM to observe the surface of rGO-PANI in greater detail. As shown in Figure 1D, the rGO-PANI composite had a three-dimensional structure characterized by many holes that increased the active surface area, leading to a dramatic increase in the cycling life of the composite.

**Figure 2:**
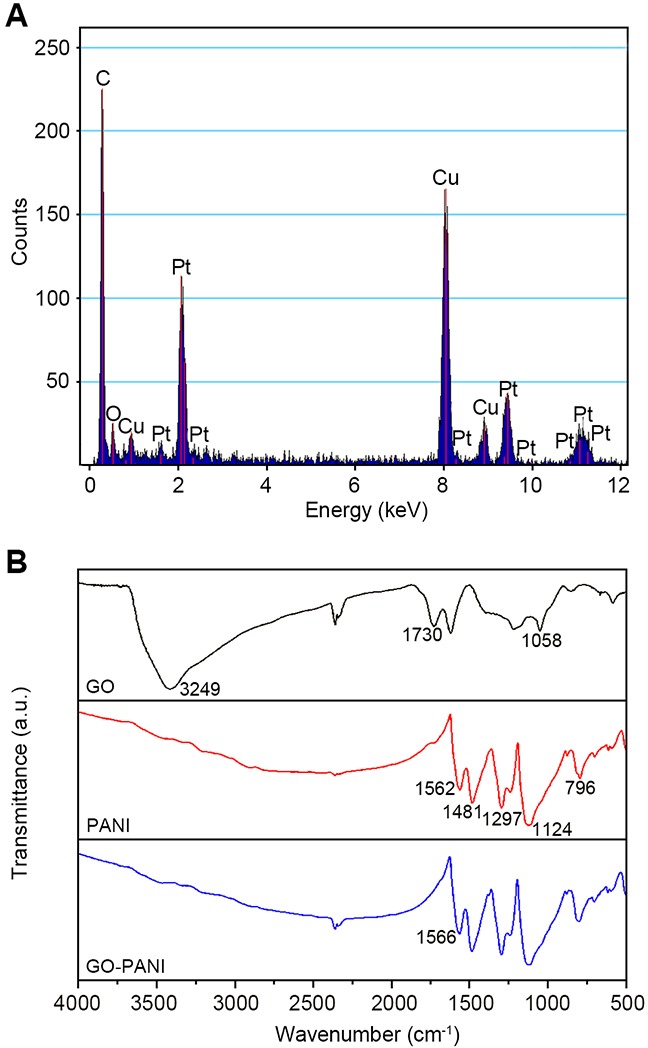
EDX analysis and FTIR spectra of the composites **(A)** EDX analysis of the rGO-PANI-PtNP composite. **(B)** FTIR spectra of GO, PANI, and GO-PANI.

EDX spectroscopy analysis was performed to confirm the elemental composition of the rGO-PANI-PtNP composite (Figure 2A). The resulting spectrum exhibited peaks corresponding to Pt and C from PtNPs and graphene, respectively, and peaks associated with Cu and O are a result of the substrate (Figure 2A). Fourier transform infrared spectroscopy (FTIR) spectra for GO, PANI, and GO-PANI were also collected in order to characterize the interaction between PANI and GO (Figure 2B). In the GO spectrum, three characteristic peaks are visible at approximately 3429, 1730, and 1058 cm^-1^, corresponding to the hydroxyl, carboxyl, and epoxide groups in GO, respectively (Figure 2B). In the PANI spectrum (Figure 2B), intense peaks at 1562 and 1481 cm^-1^ correspond to the quinoidal and benzenoid structures of PANI. Peaks at 1297 and 1124 cm^-1^ were attributed to the C-N stretching vibration in the secondary structure of the aromatic amine. The peak at 796 cm^-1^ was due to the vibration of the C-H bond out of the plane of benzene. The FTIR spectrum of the GO-PANI composite was similar to that of PANI, confirming that the GO surface was completely covered by PANI (Figure 2B). Moreover, the peak at 1562 cm^-1^ in the PANI spectrum shifted to 1566 cm^-1^ in the GO-PANI spectrum, which was attributed to the interaction between the carboxyl group of GO and the N atom of PANI. These spectra confirmed the successful preparation of both PANI and GO-PANI.

### Electrocatalytic activities of the modified electrodes

**Figure 3:**
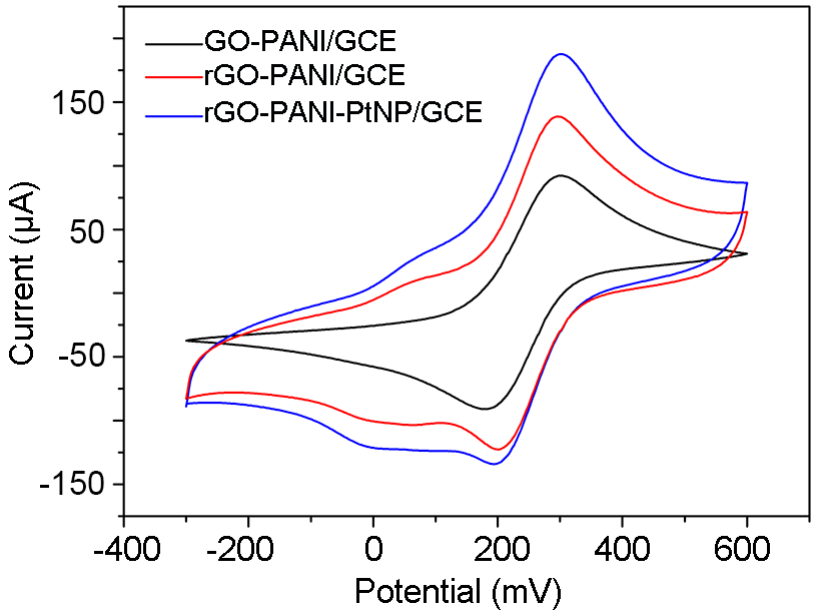
CV profiles of various electrodes recorded in 0.1 M KCl containing 10 mM [Fe(CN)_6_]^3+^ at a scan rate of 50 mV/s

We then performed cyclic voltammetry (CV) to investigate the electrical activities of GO-PANI/GCE, rGO-PANI/GCE, and rGO-PANI-PtNP/GCE. The CV experiments revealed the presence of two well-defined redox peaks for each electrode, which can be attributed to the quasi-reversible one-electron redox behavior of the ferricyanide ion (Figure 3). The values of the anodic peaks of GO-PANI/GCE, rGO-PANI/GCE, and rGO-PANI-PtNP/GCE were 92.1, 138.6, and 187.6 µA, respectively. Compared with GO-PANI/GCE, rGO-PANI/GCE showed an increase in peak current after electrochemical reduction due to the fact that rGO exhibits better electron transfer capability and conductivity than GO. The current associated with the rGO-PANI-PtNP/GCE exhibited another increase due to the electrochemical contributions of the PtNPs and synergistic effects with rGO-PANI.

The microscopic electroactive areas for each composite were calculated using the Randles–Sevcik equation *I*_p_ = 2.69×10^5^*AD*^1/2^*n*^3/2^*γ*^1/2^*C*, where *I*_p_ corresponds to the peak current, *A* represents the electroactive area of the modified electrode, *D* represents the diffusion coefficient of the molecule in the system (6.7 × 10^-6^ ± 0.02 × 10^-6^ cm^2^/s), *n* represents the number of electrons transferred (a constant), and *γ* is the scan rate. The concentration of the probe molecule is denoted by *C* and is equal to 10 mM. The calculated microscopic electroactive areas of the rGO-PANI and rGO-PANI-PtNP composites were 1.50- and 2.04-times higher than that of GO-PANI/GCE, respectively. This phenomenon is likely due to the increased surface area and superior electrical conductivity of rGO-PANI-PtNP and indicates that the rGO-PANI-PtNP nanocomposite is suitable for electrochemical detection.

**Figure 4:**
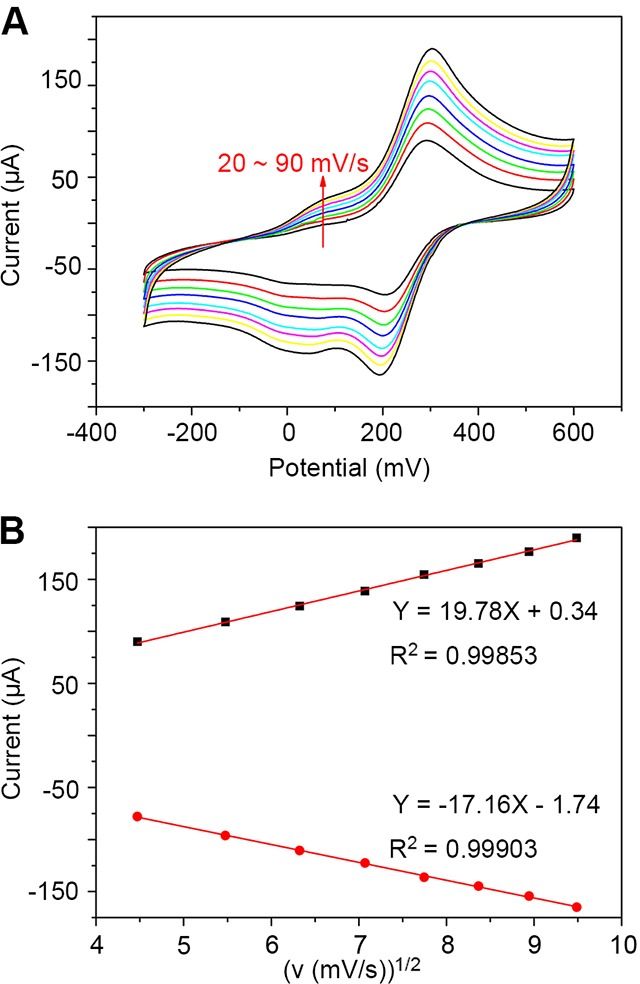
Kinetics of rGO-PANI-PtNP/GCE **(A)** Cyclic voltammograms of the sensor in 10 mM K_3_[Fe(CN)_6_] at scan rates ranging from 20 ~ 90 mV/s. **(B)** Linear fits of the oxidized peak current (*I*_pa_) and reduced peak current (*I*_pc_) versus the square root of the scan rate (*v*^1/2^).

We then examined the kinetics of rGO-PANI-PtNP/GCE by investigating the effects of scan rate on CV. The electrochemical behavior of the modified electrodes was assayed in 10 mM [Fe(CN)_6_]^3+^ at scan rates ranging from 20 ~ 90 mV/s (Figure 4A). The resulting scans show that the peak current of redox increased with increasing scan rate, and the anodic and cathodic peaks shifted towards more positive and negative potentials, respectively. Based on these results, we performed a linear fit of anodic (*I*_pa_) and cathodic (*I*_pc_) peak currents versus the square root of the scan rate (*v*^1/2^) (Figure 4B). The resulting linear equations were calculated to be *I*_pa_ = 19.78 *v* (mV/s) + 0.34 (R^2^ = 0.99853) and *I*_pc_ = 17.16 *v* (mV/s) - 1.74 (R^2^ = 0.99903). These equations indicated that the reaction of the modified electrode was a diffusion-controlled surface reaction.

**Figure 5:**
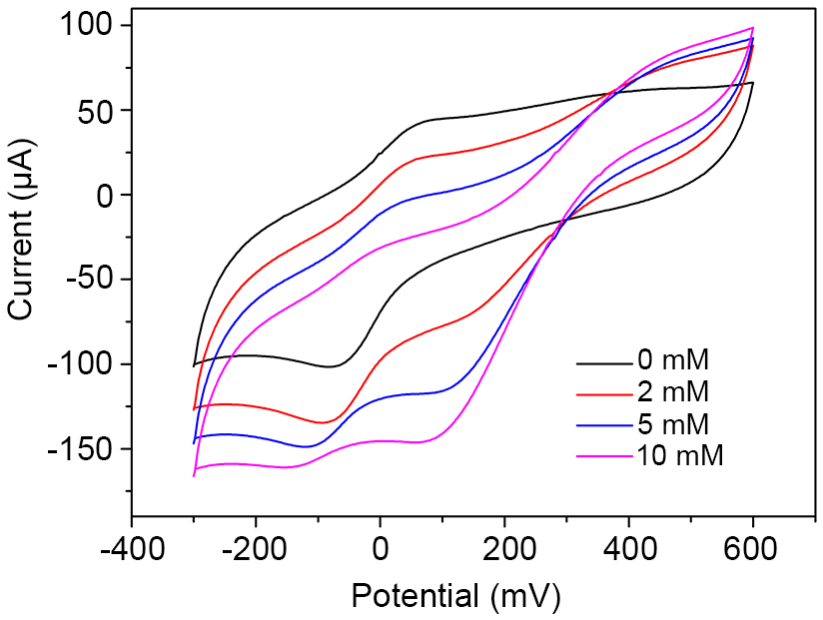
Electrocatalytic activity of rGO-PANI-PtNP/GCE towards H_2_O_2_ Cyclic voltammograms of the sensor in PB (pH 7.0) containing various concentrations of H_2_O_2_.

We next compared the CV results of rGO-PANI-PtNP/GCE in phosphate buffer (PB) and in the presence of varying concentrations of H_2_O_2_ (Figure 5). A pair of redox peaks at 30 mV and -100 mV were observed that have previously been reported to correspond to the redox transition of the leucoemeraldine form (semiconductor) to the polaronic emeraldine form (conductor) of polyaniline [20, 21]. Cathodic peaks associated with the reduction of H_2_O_2_ were enhanced with increasing concentrations of H_2_O_2_, indicating that the modified electrode exhibited excellent electrocatalytic activity towards H_2_O_2_. In the catalytic process, H_2_O_2_ reduction is initiated by dissociative adsorption of H_2_O_2_ followed by electrochemical reduction of the resultant Pt–OH [16]. The catalytic decomposition of H_2_O_2_ by PtNPs can be described by the reactions shown below:


2Pt +H2O2→k12Pt-OH4Pt-OH→k24Pt +O2+2HO2

### Optimization of experimental conditions for the fabrication of rGO-PANI-PtNP/GCE

**Figure 6:**
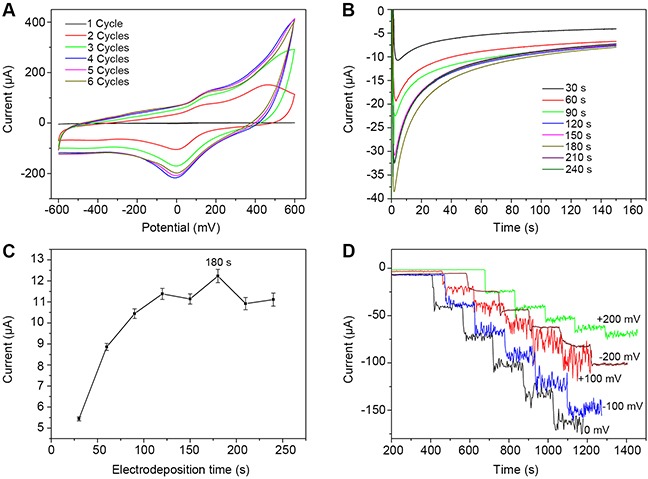
Optimization of experimental conditions for the fabrication of rGO-PANI-PtNP/GCE Effects of **(A)** reduction cycle numbers, **(B)** and **(C)** deposition time, and **(D)** applied potential on the fabrication of the sensor are shown.

Alterations in the experimental conditions used to produce modified electrodes can directly influence their electrochemical performance, leading to consequent changes in catalytic performance and sensor function. In order to optimize H_2_O_2_ detection, the effects of modifying the reduction cycle, electrodeposition time, and working potential were investigated. The effect of the reduction cycle on electrochemical performance of rGO-PANI-PtNP/GCE was analyzed by CV in 10 mM K_3_Fe(CN)_6_. The resulting spectra showed that the redox peaks associated with rGO-PANI-PtNP/GCE increased substantially after only one CV cycle (-1.5 V ~ 0 V) in N_2_-saturated PB (Figure 6A), demonstrating that GO-PANI has been reduced to rGO-PANI [15, 22]. The redox peak from rGO-PANI-PtNP/GCE began to stabilize after four reduction cycles, so we chose to use 10 cycles per experiment to ensure complete and stable reduction of GO-PANI.

The PtNPs deposited on the surface of rGO-PANI/GCE play an important role in H_2_O_2_ determination by providing a large active surface area and by enhancing the electrocatalytic activity of the electrode. Thus, the amount of PtNPs loaded on the rGO-PANI/GCE surface directly affects the function of the H_2_O_2_ sensor. To optimize the amount of PtNPs present on the electrode, the effects of varying the electrodeposition time from 30 s to 240 s were investigated. Figure 6B shows the amperometric responses associated with rGO-PANI-PtNP/GCE in the presence of 5 mM H_2_O_2_ in 0.1 M PB (pH 7.0). Figure 6C demonstrates the effect of electrodeposition time on steady-state response current after 60 s of stabilization. Results from this analysis revealed that the amperometric current associated with response to H_2_O_2_ continually increased with electrodeposition times ranging from 30 s to 180 s. After 180 s, a reduction in current response was observed, which was likely associated with excessive deposition of PtNPs that blocked electron transfer and decreased the usable surface area of the modified electrode. Therefore, based on these data, we chose 180 s as the optimal electrodeposition time for fabrication of rGO-PANI-PtNP/GCE.

We also assessed the effects of varying the applied potential from -200 mV to +200 mV on the amperometric response of rGO-PANI-PtNP/GCE to five successive additions of 1 mM H_2_O_2_ in 0.1 M PB (Figure 6D). Results from this analysis reveal that the response current increased with increasing applied potential from -200 mV to 0 mV, at which point the maximum value was reached. After this point, the current response decreased, and the signal-to-noise ratio increased. Thus, 0 mV versus an Ag/AgCl reference electrode was selected as the optimum applied potential for H_2_O_2_detection. It is important to note that this applied potential for rGO-PANI-PtNP/GCE is much smaller than that of many previously reported H_2_O_2_ amperometric sensors (Table 1). Use of a low potential can minimize the response of common interference materials and can decrease the background current, leading to improved H_2_O_2_ detection.

**Table 1:** Comparison of selected electrochemical sensors for H_2_O_2_ detection

Electrodes	Applied potential (mV)	Sensitivity (μA mM^−1^ cm^-2^)	LOD (μM)	Liner range (mM)	Refs
Pt/Cu/C/GCE	+300	69.4	12.2	up to 4	[23]
Silver nanowire	-200	0.0266	29.2	0.1-3.1	[24]
CQDs^a^/octahedral Cu_2_O	-200	130	2.8	0.005-5.3	[25]
Pt-SnO_2_@C	+500	241	0.1	0.001-0.17	[26]
Ag-Au-rGO	-400	-	1	0.1-5	[27]
PdNPs/PEDOT^b^/GCE	-400	215	2.84	0.0025-1	[28]
Pt-polypyrrole/GCE	-100	80.41	1.2	1-8	[29]
graphene/PB^c^/GCE	-50	196.6	1.9	0.02-2	[30]
rGO-PANI-PtNP/GCE	0	257.04	1.1	0.02-8	this study

^a^ carbon quantum dots; ^b^ poly(3,4-ethylenedioxythiophene); ^c^ prussian blue.

### Amperometric responses to H_2_O_2_

**Figure 7:**
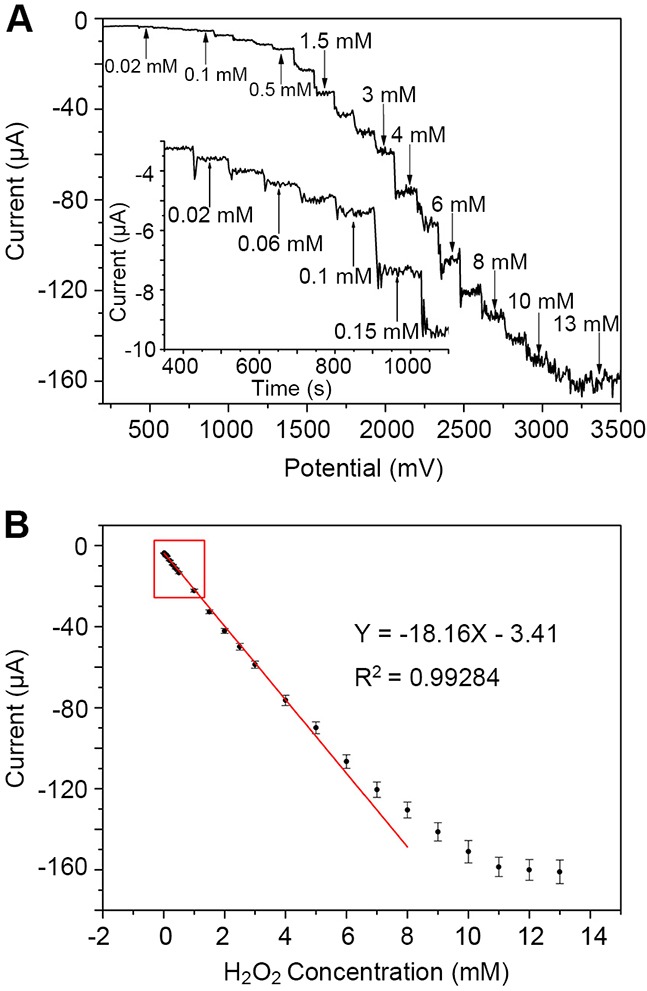
Electrochemical detection of H_2_O_2_ by rGO-PANI-PtNP/GCE **(A)** Amperometric response of the sensor to successive additions of H_2_O_2_ in 0.1 M PB (pH 7.0) at 0 mV. Insert: Amplification of the current/time curve at lower concentrations of H_2_O_2_. **(B)** Calibration curve for H_2_O_2_ sensing. Error bars represent the mean ± standard deviation (n = 5).

We next examined the amperometric responses of rGO-PANI-PtNP/GCE to successive addition of different concentrations of H_2_O_2_ at a potential of 0 mV. As shown in Figure 7A, a typical stepwise increase in reduction current was observed in response to H_2_O_2_. Each reduction current response exhibited a well-defined increase as the concentration of H_2_O_2_ increased, reaching a steady-state value with a rapid response time (less than 4 s to achieving ≥ 95% steady-state current). The inset shows the current response at lower concentrations of H_2_O_2_ (0.02 ~ 0.15 mM).

Using data from five replicates, we fitted the calibration curve displayed in Figure 7B. Based on the curve fit, we calculated the linear range of the H_2_O_2_ sensor to be from 0.02 to 8 mM with a correlation coefficient of 0.996 and a relative standard deviation ranging from 2.1 to 3.4% (n = 5). The equation associated with the linear regression of the sensor was *I* (μA) = -18.16*C* (mM) - 3.41. The sensitivity and the limit of detection (LOD) were 257.04 μA mM^-1^ cm^-2^ (18.16 μA mM^-1^) and 1.1 µM (signal-to-noise ratio of 3), respectively. This H_2_O_2_ sensor exhibits superior performance with respect to linear range, sensitivity, and LOD in comparison to other reported H_2_O_2_ sensors (Table 1). Thus, the rGO-PANI-PtNP/GCE sensor is highly applicable for H_2_O_2_ detection.

Performance of rGO-PANI-PtNP/GCE in real sample analysis was investigated by analyzing H_2_O_2_ levels and evaluating the percent recovery in fetal bovine serum (FBS) added with different amount of H_2_O_2_ (Table 2). In the range of 97.5 to 103% recovery, the relative standard deviation (RSD) ranged from 2.6 to 3.2%. These results demonstrate the applicability of the H_2_O_2_ sensor for real-sample determination.

**Table 2:** Quantification of H_2_O_2_ in FBS containing different concentrations of H_2_O_2_

Actual H_2_O_2_ (mM)	Measured H_2_O_2_ (mM)	RSD (%, n = 6)	Recovery (%)
0.1	0.103	3.2	103
0.4	0.39	3.8	97.5
1	0.98	4.1	98
3	3.09	4.2	103
5	5.12	4.5	102.4

### Selectivity, stability, and reproducibility of rGO-PANI-PtNP/GCE

**Figure 8:**
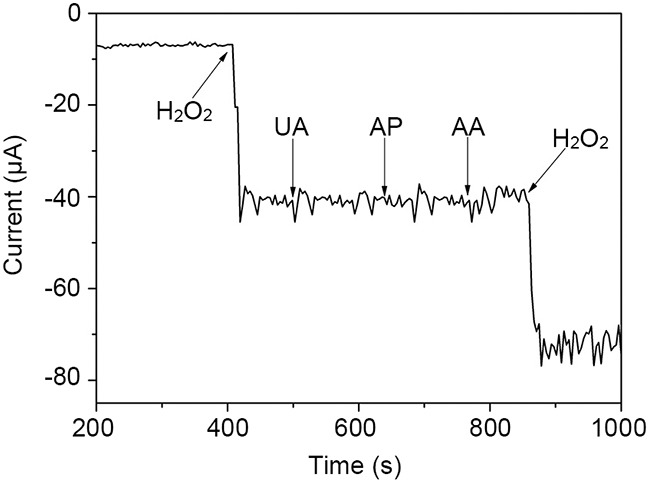
Amperometric responses of rGO-PANI-PtNP/GCE to 1 mM H_2_O_2_, 0.5 mM UA, 0.15 mM AP, 0.15 mM AA, and 1 mM H_2_O_2_ in PB at an applied potential of 0 mV

Uric acid (UA), acetaminophen (AP), and ascorbic acid (AA) are three of the most common electroactive contaminants that cause serious interference with electrochemical detection of H_2_O_2_ in biological samples. The concentrations of UA, AP, and AA in human blood are approximately 0.33 mM, 0.13 mM, and 0.125 mM, respectively. Therefore, in order to evaluate the selectivity and interference levels for these components with respect to rGO-PANI-PtNP/GCE, we tested the amperometric responses to 0.5 mM UA, 0.15 mM AP, and 0.15 mM AA at a potential of 0 mV. We found that the current responses associated with UA, AP, and AA were negligible compared with the amplitude of the current for 1 mM H_2_O_2_ (Figure 8). These data indicate that rGO-PANI-PtNP/GCE can be used for highly selective electrochemical detection of H_2_O_2_ in biological samples. The superior selectivity and lack of interference for the modified electrode is mainly due to its low working potential value of 0 mV.

Reproducibility and long-term stability of the sensor are also major parameters that can affect the applicability of H_2_O_2_ sensors. Reproducibility of the novel sensor was analyzed by measuring the responses of five independently produced electrodes to 1 mM H_2_O_2_, yielding a RSD of 3.4%. Long-term stability was evaluated by measuring the response elicited by exposure to 1 mM H_2_O_2_ every week for one month. rGO-PANI-PtNP retained 88% of its initial current response for H_2_O_2_. Thus, the rGO-PANI-PtNP electrode exhibited acceptable long-term stability.

## CONCLUSION

In summary, we found that synthesis of PANI using GO as a template resulted in a composite that had satisfactory water solubility and stability. We then used electrochemical methods to prepare an rGO-PANI-PtNP nanocomposite on the surface of GCE. The resulting rGO-PANI-PtNP/GCE exhibited excellent electrochemical performance and was successfully applied as a novel, non-enzymatic H_2_O_2_ sensor. The sensor exhibited superb H_2_O_2_ detection with reproducibility and sensitivity values better than most previously described H_2_O_2_ sensors. Thus, we propose that this modified electrode can act as a sensor for the analysis of H_2_O_2_ in a wide variety of samples.

## MATERIALS AND METHODS

### Chemicals and reagents

GO was purchased from Xfnano Materials Tech (Nanjing, China). H_2_O_2_ was obtained from Damao Chemical Reagent (Tianjin, China). Aniline, potassium hexachloroplatinate (KPtCl_6_), ammonium peroxydisulfate, ascorbic acid, uric acid, and acetaminophen were purchased from Sigma-Aldrich (St. Louis, MO, USA). Phosphate buffer (PB, 0.1 M, pH 7.0) was prepared using Na_2_HPO_4_ and NaH_2_PO_4_. Doubly distilled water was used to prepare all aqueous solutions. All experiments were performed at room temperature.

### Analytical methods

Electrochemical measurements were performed using a EG&G 283 Potentiostat-Galvanostat electrochemical workstationequipped with the M270 software (Ametek, Berwyn, PA, USA). An electrochemical cell connected to a standard three-electrode system was employed for all electrochemical measurements. An Ag/AgCl (saturated KCl) electrode served as the reference electrode. A 3-mm-diameter modified GCE and a platinum wire (1 mm diameter) were used as the working electrode and the counter electrode, respectively. For steady-state amperometric experiments, the potential was set to 0 mV in PBS with slight stirring. Scanning electron microscopy (SEM) and transmission electron microscopy (TEM) images were captured using a Quanta-200 field emission microscope (FEI, Hillsboro, OR, USA) and a Tecnai G2 F20 instrument equipped with energy-dispersive X-ray spectroscopy (Philips, Amsterdam, Netherlands), respectively. Fourier transform infrared spectroscopy (FTIR) spectra were collected on a Tensor-37 instrument (Bruker, Billerica, MA, USA).

### Fabrication of homogenous GO-PANI composite

Homogenous GO-PANI composite was prepared according to previously reported methods with slight modifications [15]. Briefly, purified aniline was dissolved in 1 M HCl at a concentration of 0.3 M. GO (1:9 molar ratio GO to aniline) was dissolved in the resulting solution by sonicating in a bath for 1 hr. A solution of ammonium peroxydisulfate in 1 M HCl (1:4 molar ratio ammonium peroxydisulfate to aniline) was rapidly poured to the mixture with vigorous stirring at room temperature. Polymerization of aniline initiated after 5 min, and the solution turned green. The mixture was allowed to stir at room temperature overnight, and then the dark homogeneous solution was centrifuged and washed repeatedly using a mixture of doubly distilled water, hexane, and ethanol until the pH was neutralized. The solution was then dried in drying oven to obtain the GO-PANI composite. GO-PANI was then re-dissolved in PB (5 mg/mL) by sonicating in a bath for subsequent applications. PANI was prepared using the same method, except graphene oxide was not added to the solution.

### Electrode modification

A bare GCE was first carefully polished with alumina powders (0.3 and 0.05 μm) to remove oxide layers on the surface of the GCE. After polishing, the GCE was ultrasonically cleaned using double distilled water and ethanol for 20 min to remove any physically adsorbed substances. The electrode was then immediately dried under nitrogen gas. The electrode was coated with GO-PANI film by placing 8 μL of GO-PANI suspension (5 mg/mL) onto the surface of the GCE, followed by drying at room temperature. GO-PANI/GCE was electrochemically reduced to rGO-PANI/GCE by scanning from -1.5 V to 0 V (10 cycles) using cyclic voltammetry (CV) in N_2_-saturated PB. rGO-PANI/GCE was then subjected to electrodeposition in 30 mL of 10 mM KPtCl_6_ solution (in 0.1 M PB) for 180 sec at -200 mV to obtain the rGO-PANI-PtNP/GCE.
